# Tetra­aqua­bis­{5-[4-(imidazol-1-yl-κ*N* ^3^)phen­yl]tetra­zolido}manganese(II)

**DOI:** 10.1107/S1600536811047428

**Published:** 2011-11-12

**Authors:** Xiao-Chun Cheng

**Affiliations:** a Faculty of Life Science and Chemical Engineering, Huaiyin Institute of Technology, Huaian 223003, People’s Republic of China

## Abstract

In the title complex, [Mn(C_10_H_7_N_6_)_2_(H_2_O)_4_], the Mn^2+^ cation is located on a twofold rotation axis and is coordinated by two N atoms from two 5-[4-(imidazol-1-yl)phen­yl]tetra­zolide ligands and four O atoms from four water mol­ecules, displaying a distorted MnN_2_O_4_ octa­hedral geometry. The crystal structure is stabilized by intermolecular O—H⋯N hydrogen bonds involving the coordinated water mol­ecules and the N atoms of the tetra­zolide group.

## Related literature

For related structures, see: Huang *et al.* (2009 ▶).
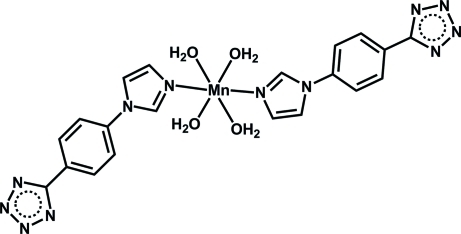


## Experimental

### 

#### Crystal data

[Mn(C_10_H_7_N_6_)_2_(H_2_O)_4_]
*M* *_r_* = 549.44
Triclinic, 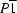

*a* = 8.415 (3) Å
*b* = 8.458 (3) Å
*c* = 8.722 (3) Å
α = 80.758 (5)°
β = 75.880 (4)°
γ = 88.791 (5)°
*V* = 594.1 (4) Å^3^
*Z* = 1
Mo *K*α radiation
μ = 0.61 mm^−1^
*T* = 293 K
0.20 × 0.20 × 0.20 mm

#### Data collection

Bruker SMART APEXII CCD diffractometer
Absorption correction: multi-scan (*SADABS*; Sheldrick, 1996 ▶) *T*_min_ = 0.888, *T*_max_ = 0.888
3143 measured reflections
2198 independent reflections
2027 reflections with *I* > 2σ(*I*)
*R*_int_ = 0.014

#### Refinement

*R*[*F* ^2^ > 2σ(*F* ^2^)] = 0.033
*wR*(*F* ^2^) = 0.088
*S* = 1.07
2198 reflections
169 parameters
H-atom parameters constrained
Δρ_max_ = 0.23 e Å^−3^
Δρ_min_ = −0.30 e Å^−3^

### 

Data collection: *APEX2* (Bruker, 2008 ▶); cell refinement: *SAINT* (Bruker, 2008 ▶); data reduction: *SAINT*; program(s) used to solve structure: *SHELXS97* (Sheldrick, 2008 ▶); program(s) used to refine structure: *SHELXL97* (Sheldrick, 2008 ▶); molecular graphics: *DIAMOND* (Brandenburg, 2000 ▶); software used to prepare material for publication: *SHELXTL* (Sheldrick, 2008 ▶).

## Supplementary Material

Crystal structure: contains datablock(s) I, global. DOI: 10.1107/S1600536811047428/pv2481sup1.cif

Structure factors: contains datablock(s) I. DOI: 10.1107/S1600536811047428/pv2481Isup2.hkl

Supplementary material file. DOI: 10.1107/S1600536811047428/pv2481Isup4.cdx

Additional supplementary materials:  crystallographic information; 3D view; checkCIF report

## Figures and Tables

**Table 1:** Hydrogen-bond geometry (Å, °)

*D*—H⋯*A*	*D*—H	H⋯*A*	*D*⋯*A*	*D*—H⋯*A*
O2—H2*W*⋯N5^i^	0.85	2.00	2.796 (2)	155
O2—H2*WA*⋯N6^ii^	0.82	2.05	2.844 (2)	161
O1—H1*W*⋯N4^i^	0.92	1.93	2.819 (2)	163
O1—H1*WA*⋯N3^iii^	0.85	1.92	2.769 (2)	175

Symmetry codes: (i) 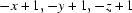; (ii) 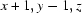; (iii) 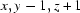.

**Fig. 1.**
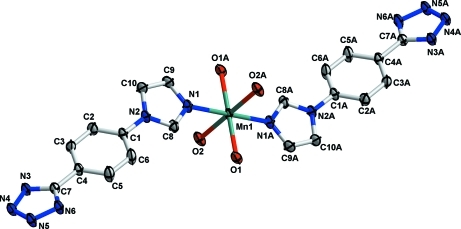
: The coordination environment of manganese ion in the title complex with the ellipsoids drawn at the 30% probability level. The hydrogen atoms have been omitted for clarity. Symmetry code: A = -x + 2, -y, -z+2.

## supplementary crystallographic information

### Comment

Multidentate ligand, 1-(5-tetrazolyl)-4-(imidazol-1-yl)benzene, may be used to synthesize complexes for its variable coordination modes. Herein, we report the crystal structure of the title complex wherein the Mn ion is located on a two fold rotation axis and is coordinated by two N atoms from two ligand molecules and four O atoms from four coordinated water molecules, displaying a distorted MnN_2_O_4_ octahedral geometry (Fig. 1). The ligand displays a monodentate coordinating mode and acts as counteranion due to the deprotonation of tetrazolyl group. In the crystal structure, there exist O—H···N hydrogen bonds (Table 1). Coordinated water molecules and N atoms of tetrazolyl group as donor or acceptor play very important role in the formation of these hydrogen bonds and stabilize the crystal structure.

### Experimental

Reaction mixture of manganese perchlorate hexahydrate (72.3 mg, 0.2 mmol), 1-(5-tetrazolyl)-4-(imidazol-1-yl)benzene (21.2 mg, 0.1 mmol), and potassium hydroxide (5.61 mg, 0.1 mmol) in 12 ml H_2_O was sealed in a 16 ml Teflon-lined stainless steel container and heated to 393 K for 3 days. After cooling the stainless steel container to the room temperature, colorless block crystals of the title complex were obtained.

### Refinement

The hydrogen atoms in all C atoms were located in geometrically idealized positions and constrained to ride on their parent atoms, with C—H = 0.93 Å and *U*_iso_(H) = 1.2*U*_eq_(C). The hydrogen atoms of water molecules were found from a difference Fourier map and fixed at those positions with *U*_iso_(H) = 1.2*U*_eq_(O).

### Crystal data

**Table:**

[Mn(C_10_H_7_N_6_)_2_(H_2_O)_4_]	*Z* = 1
*M**_r_* = 549.44	*F*(000) = 283
Triclinic, *P*1	*D*_x_ = 1.536 Mg m^−^^3^
Hall symbol: -P 1	Mo *K*α radiation, λ = 0.71073 Å
*a* = 8.415 (3) Å	Cell parameters from 2041 reflections
*b* = 8.458 (3) Å	θ = 2.4–28.3°
*c* = 8.722 (3) Å	µ = 0.61 mm^−^^1^
α = 80.758 (5)°	*T* = 293 K
β = 75.880 (4)°	Block, colorless
γ = 88.791 (5)°	0.20 × 0.20 × 0.20 mm
*V* = 594.1 (4) Å^3^	

### Data collection

**Table:**

Bruker SMART APEXII CCD diffractometer	2198 independent reflections
Radiation source: fine-focus sealed tube	2027 reflections with *I* > 2σ(*I*)
graphite	*R*_int_ = 0.014
φ and ω scans	θ_max_ = 25.6°, θ_min_ = 2.4°
Absorption correction: multi-scan (*SADABS*; Sheldrick, 1996)	*h* = −9→10
*T*_min_ = 0.888, *T*_max_ = 0.888	*k* = −5→10
3143 measured reflections	*l* = −10→10

### Refinement

**Table:**

Refinement on *F*^2^	Primary atom site location: structure-invariant direct methods
Least-squares matrix: full	Secondary atom site location: difference Fourier map
*R*[*F*^2^ > 2σ(*F*^2^)] = 0.033	Hydrogen site location: inferred from neighbouring sites
*wR*(*F*^2^) = 0.088	H-atom parameters constrained
*S* = 1.07	*w* = 1/[σ^2^(*F*_o_^2^) + (0.0442*P*)^2^ + 0.2267*P*] where *P* = (*F*_o_^2^ + 2*F*_c_^2^)/3
2198 reflections	(Δ/σ)_max_ < 0.001
169 parameters	Δρ_max_ = 0.23 e Å^−^^3^
0 restraints	Δρ_min_ = −0.30 e Å^−^^3^

### Special details

**Table:**

Geometry. All e.s.d.'s (except the e.s.d. in the dihedral angle between two l.s. planes) are estimated using the full covariance matrix. The cell e.s.d.'s are taken into account individually in the estimation of e.s.d.'s in distances, angles and torsion angles; correlations between e.s.d.'s in cell parameters are only used when they are defined by crystal symmetry. An approximate (isotropic) treatment of cell e.s.d.'s is used for estimating e.s.d.'s involving l.s. planes.
Refinement. Refinement of *F*^2^ against ALL reflections. The weighted *R*-factor *wR* and goodness of fit *S* are based on *F*^2^, conventional *R*-factors *R* are based on *F*, with *F* set to zero for negative *F*^2^. The threshold expression of *F*^2^ > σ(*F*^2^) is used only for calculating *R*-factors(gt) *etc*. and is not relevant to the choice of reflections for refinement. *R*-factors based on *F*^2^ are statistically about twice as large as those based on *F*, and *R*- factors based on ALL data will be even larger.

### Fractional atomic coordinates and isotropic or equivalent isotropic displacement parameters (Å^2^)

**Table:**

	*x*	*y*	*z*	*U*_iso_*/*U*_eq_	
C1	0.7451 (2)	0.5606 (2)	0.7075 (2)	0.0361 (4)	
C2	0.8080 (3)	0.6654 (3)	0.5691 (3)	0.0486 (5)	
H2	0.9208	0.6794	0.5304	0.058*	
C3	0.7036 (2)	0.7498 (3)	0.4877 (3)	0.0443 (5)	
H3	0.7472	0.8196	0.3934	0.053*	
C4	0.5364 (2)	0.7327 (2)	0.5431 (2)	0.0324 (4)	
C5	0.4748 (3)	0.6291 (3)	0.6838 (3)	0.0557 (6)	
H5	0.3620	0.6169	0.7239	0.067*	
C6	0.5787 (3)	0.5430 (3)	0.7659 (3)	0.0550 (6)	
H6	0.5357	0.4734	0.8605	0.066*	
C7	0.4287 (2)	0.8208 (2)	0.4509 (2)	0.0310 (4)	
C8	0.8260 (3)	0.3192 (2)	0.8712 (2)	0.0396 (4)	
H8	0.7257	0.2651	0.8954	0.048*	
C9	1.0742 (3)	0.3767 (3)	0.8576 (3)	0.0541 (6)	
H9	1.1804	0.3691	0.8713	0.065*	
C10	1.0145 (3)	0.5054 (3)	0.7786 (3)	0.0583 (7)	
H10	1.0707	0.6004	0.7280	0.070*	
Mn1	1.0000	0.0000	1.0000	0.02857 (14)	
N1	0.9560 (2)	0.25905 (19)	0.9145 (2)	0.0393 (4)	
N2	0.8541 (2)	0.46819 (19)	0.7876 (2)	0.0382 (4)	
N3	0.48043 (19)	0.8941 (2)	0.29971 (18)	0.0366 (4)	
N4	0.3458 (2)	0.9572 (2)	0.25971 (18)	0.0392 (4)	
N5	0.21984 (19)	0.9230 (2)	0.38155 (19)	0.0413 (4)	
N6	0.26763 (19)	0.8368 (2)	0.50521 (18)	0.0386 (4)	
O1	0.74178 (16)	−0.05589 (19)	1.03254 (16)	0.0461 (4)	
H1WA	0.6635	−0.0665	1.1168	0.055*	
H1W	0.6996	−0.0410	0.9438	0.055*	
O2	1.03428 (16)	−0.04640 (18)	0.75458 (15)	0.0415 (3)	
H2W	0.9801	−0.0074	0.6872	0.050*	
H2WA	1.1108	−0.0900	0.6996	0.050*	

### Atomic displacement parameters (Å^2^)

**Table:**

	*U*^11^	*U*^22^	*U*^33^	*U*^12^	*U*^13^	*U*^23^
C1	0.0377 (10)	0.0331 (10)	0.0391 (11)	0.0082 (8)	−0.0168 (8)	0.0006 (8)
C2	0.0297 (10)	0.0528 (13)	0.0558 (14)	0.0005 (9)	−0.0126 (9)	0.0164 (10)
C3	0.0347 (10)	0.0464 (12)	0.0438 (12)	−0.0013 (9)	−0.0098 (9)	0.0166 (9)
C4	0.0314 (9)	0.0353 (9)	0.0294 (9)	0.0071 (7)	−0.0086 (7)	−0.0006 (7)
C5	0.0304 (11)	0.0787 (17)	0.0436 (12)	0.0086 (10)	−0.0024 (9)	0.0214 (11)
C6	0.0427 (12)	0.0678 (15)	0.0411 (12)	0.0094 (11)	−0.0057 (10)	0.0229 (11)
C7	0.0281 (9)	0.0377 (9)	0.0259 (9)	0.0050 (7)	−0.0064 (7)	−0.0018 (7)
C8	0.0394 (10)	0.0348 (10)	0.0434 (11)	0.0055 (8)	−0.0155 (9)	0.0049 (8)
C9	0.0471 (13)	0.0433 (12)	0.0767 (17)	0.0004 (10)	−0.0358 (12)	0.0085 (11)
C10	0.0505 (13)	0.0410 (12)	0.0866 (19)	−0.0056 (10)	−0.0392 (13)	0.0158 (12)
Mn1	0.0232 (2)	0.0344 (2)	0.0247 (2)	0.00559 (15)	−0.00588 (15)	0.00465 (15)
N1	0.0427 (9)	0.0342 (8)	0.0419 (9)	0.0069 (7)	−0.0179 (8)	0.0015 (7)
N2	0.0398 (9)	0.0337 (8)	0.0434 (9)	0.0063 (7)	−0.0202 (8)	0.0022 (7)
N3	0.0278 (8)	0.0523 (10)	0.0270 (8)	0.0062 (7)	−0.0069 (6)	0.0015 (7)
N4	0.0318 (8)	0.0579 (11)	0.0259 (8)	0.0081 (7)	−0.0097 (7)	0.0019 (7)
N5	0.0303 (8)	0.0630 (11)	0.0277 (8)	0.0126 (8)	−0.0077 (7)	0.0008 (8)
N6	0.0294 (8)	0.0560 (10)	0.0268 (8)	0.0117 (7)	−0.0060 (6)	0.0015 (7)
O1	0.0231 (6)	0.0790 (11)	0.0296 (7)	0.0009 (6)	−0.0052 (5)	0.0090 (7)
O2	0.0329 (7)	0.0649 (9)	0.0247 (7)	0.0167 (6)	−0.0066 (5)	−0.0037 (6)

### Geometric parameters (Å, °)

**Table:**

C1—C6	1.371 (3)	C9—N1	1.367 (3)
C1—C2	1.374 (3)	C9—H9	0.9300
C1—N2	1.431 (2)	C10—N2	1.373 (3)
C2—C3	1.379 (3)	C10—H10	0.9300
C2—H2	0.9300	Mn1—O1	2.1737 (15)
C3—C4	1.374 (3)	Mn1—O1^i^	2.1737 (15)
C3—H3	0.9300	Mn1—O2	2.1870 (15)
C4—C5	1.381 (3)	Mn1—O2^i^	2.1870 (15)
C4—C7	1.469 (2)	Mn1—N1	2.2514 (17)
C5—C6	1.385 (3)	Mn1—N1^i^	2.2514 (17)
C5—H5	0.9300	N3—N4	1.340 (2)
C6—H6	0.9300	N4—N5	1.305 (2)
C7—N6	1.334 (2)	N5—N6	1.343 (2)
C7—N3	1.334 (2)	O1—H1WA	0.8528
C8—N1	1.310 (2)	O1—H1W	0.9171
C8—N2	1.345 (3)	O2—H2W	0.8511
C8—H8	0.9300	O2—H2WA	0.8226
C9—C10	1.348 (3)		
			
C6—C1—C2	119.82 (18)	O1—Mn1—O2	86.80 (5)
C6—C1—N2	120.54 (18)	O1^i^—Mn1—O2	93.20 (5)
C2—C1—N2	119.62 (18)	O1—Mn1—O2^i^	93.20 (5)
C1—C2—C3	119.91 (19)	O1^i^—Mn1—O2^i^	86.80 (5)
C1—C2—H2	120.0	O2—Mn1—O2^i^	180.0
C3—C2—H2	120.0	O1—Mn1—N1	90.17 (6)
C4—C3—C2	121.27 (19)	O1^i^—Mn1—N1	89.83 (6)
C4—C3—H3	119.4	O2—Mn1—N1	89.07 (6)
C2—C3—H3	119.4	O2^i^—Mn1—N1	90.93 (6)
C3—C4—C5	118.23 (18)	O1—Mn1—N1^i^	89.83 (6)
C3—C4—C7	119.87 (17)	O1^i^—Mn1—N1^i^	90.17 (6)
C5—C4—C7	121.88 (17)	O2—Mn1—N1^i^	90.93 (6)
C4—C5—C6	120.9 (2)	O2^i^—Mn1—N1^i^	89.07 (6)
C4—C5—H5	119.5	N1—Mn1—N1^i^	180.0
C6—C5—H5	119.5	C8—N1—C9	105.09 (17)
C1—C6—C5	119.8 (2)	C8—N1—Mn1	127.28 (14)
C1—C6—H6	120.1	C9—N1—Mn1	125.60 (14)
C5—C6—H6	120.1	C8—N2—C10	106.15 (16)
N6—C7—N3	111.17 (16)	C8—N2—C1	126.58 (17)
N6—C7—C4	125.02 (17)	C10—N2—C1	126.85 (17)
N3—C7—C4	123.81 (16)	C7—N3—N4	105.15 (15)
N1—C8—N2	112.29 (19)	N5—N4—N3	109.18 (15)
N1—C8—H8	123.9	N4—N5—N6	109.87 (15)
N2—C8—H8	123.9	C7—N6—N5	104.64 (15)
C10—C9—N1	110.2 (2)	Mn1—O1—H1WA	130.2
C10—C9—H9	124.9	Mn1—O1—H1W	118.1
N1—C9—H9	124.9	H1WA—O1—H1W	109.5
C9—C10—N2	106.3 (2)	Mn1—O2—H2W	127.1
C9—C10—H10	126.8	Mn1—O2—H2WA	129.2
N2—C10—H10	126.8	H2W—O2—H2WA	102.8
O1—Mn1—O1^i^	180.0		
			
C6—C1—C2—C3	−1.4 (4)	O2^i^—Mn1—N1—C8	−107.15 (18)
N2—C1—C2—C3	177.3 (2)	N1^i^—Mn1—N1—C8	144 (100)
C1—C2—C3—C4	0.7 (4)	O1—Mn1—N1—C9	−175.28 (19)
C2—C3—C4—C5	0.4 (3)	O1^i^—Mn1—N1—C9	4.72 (19)
C2—C3—C4—C7	−178.2 (2)	O2—Mn1—N1—C9	−88.48 (19)
C3—C4—C5—C6	−0.8 (4)	O2^i^—Mn1—N1—C9	91.52 (19)
C7—C4—C5—C6	177.7 (2)	N1^i^—Mn1—N1—C9	−17 (100)
C2—C1—C6—C5	1.0 (4)	N1—C8—N2—C10	−0.5 (3)
N2—C1—C6—C5	−177.7 (2)	N1—C8—N2—C1	172.43 (18)
C4—C5—C6—C1	0.1 (4)	C9—C10—N2—C8	−0.1 (3)
C3—C4—C7—N6	−167.3 (2)	C9—C10—N2—C1	−173.0 (2)
C5—C4—C7—N6	14.2 (3)	C6—C1—N2—C8	31.4 (3)
C3—C4—C7—N3	13.6 (3)	C2—C1—N2—C8	−147.2 (2)
C5—C4—C7—N3	−164.9 (2)	C6—C1—N2—C10	−157.1 (2)
N1—C9—C10—N2	0.6 (3)	C2—C1—N2—C10	24.2 (3)
N2—C8—N1—C9	0.8 (3)	N6—C7—N3—N4	−0.1 (2)
N2—C8—N1—Mn1	−163.53 (14)	C4—C7—N3—N4	179.14 (17)
C10—C9—N1—C8	−0.9 (3)	C7—N3—N4—N5	0.0 (2)
C10—C9—N1—Mn1	163.81 (18)	N3—N4—N5—N6	0.1 (2)
O1—Mn1—N1—C8	−13.94 (18)	N3—C7—N6—N5	0.2 (2)
O1^i^—Mn1—N1—C8	166.06 (18)	C4—C7—N6—N5	−179.05 (18)
O2—Mn1—N1—C8	72.85 (18)	N4—N5—N6—C7	−0.2 (2)

Symmetry codes: (i) −*x*+2, −*y*, −*z*+2.

### Hydrogen-bond geometry (Å, °)

**Table:**

*D*—H···*A*	*D*—H	H···*A*	*D*···*A*	*D*—H···*A*
O2—H2W···N5^ii^	0.85	2.00	2.796 (2)	155.
O2—H2WA···N6^iii^	0.82	2.05	2.844 (2)	161.
O1—H1W···N4^ii^	0.92	1.93	2.819 (2)	163.
O1—H1WA···N3^iv^	0.85	1.92	2.769 (2)	175.

Symmetry codes: (ii) −*x*+1, −*y*+1, −*z*+1; (iii) *x*+1, *y*−1, *z*; (iv) *x*, *y*−1, *z*+1.
